# New Chloramphenicol Derivatives with a Modified Dichloroacetyl Tail as Potential Antimicrobial Agents

**DOI:** 10.3390/antibiotics10040394

**Published:** 2021-04-06

**Authors:** Artemis Tsirogianni, Georgia G. Kournoutou, Anthony Bougas, Eleni Poulou-Sidiropoulou, George Dinos, Constantinos M. Athanassopoulos

**Affiliations:** 1 Synthetic Organic Chemistry Laboratory, Department of Chemistry, University of Patras, 26504 Patras, Greece; chem3539@upnet.gr; 2 Department of Biochemistry, School of Medicine, University of Patras, 26504 Patras, Greece; gkurnutu@upatras.gr (G.G.K.); bogant@upatras.gr (A.B.); up1002533@upnet.gr (E.P.-S.)

**Keywords:** antibiotics, chloramphenicol, ribosome, peptidyl transferase, antibiotic resistance

## Abstract

To combat the dangerously increasing pathogenic resistance to antibiotics, we developed new pharmacophores by chemically modifying a known antibiotic, which remains to this day the most familiar and productive way for novel antibiotic development. We used as a starting material the chloramphenicol base, which is the free amine group counterpart of the known chloramphenicol molecule antibiotic upon removal of its dichloroacetyl tail. To this free amine group, we tethered alpha- and beta-amino acids, mainly glycine, lysine, histidine, ornithine and/or beta-alanine. Furthermore, we introduced additional modifications to the newly incorporated amine groups either with protecting groups triphenylmethyl- (Trt) and tert-butoxycarbonyl- (Boc) or with the dichloroacetic group found also in the chloramphenicol molecule. The antimicrobial activity of all compounds was tested both in vivo and in vitro, and according to the results, the bis-dichloroacetyl derivative of ornithine displayed the highest antimicrobial activity both in vivo and in vitro and seems to be a dynamic new pharmacophore with room for further modification and development.

## 1. Introduction

Antibiotics changed the course of therapeutics and have saved countless lives worldwide since their first clinical introduction [1]. Infectious diseases that could not be previously treated and their catastrophic effects are now easily controlled. However, shortly after their introduction, resistance began to emerge rapidly, and now multidrug-resistant bacteria have become a major concern for these infections’ treatment [2]. It has become evident that novel anti-infective agents with better efficiency and/or alternative modes of action are urgently required to battle the ever-evolving multidrug-resistant bacteria. Many research groups worldwide are now devoted to solving this crisis either looking for new natural compounds or derivatizing known antibiotics [3,4]. Following the second strategy, we present here new derivatives of chloramphenicol (CAM) with important antimicrobial activity, which show great promise as new scaffolds for further development of superior antimicrobial agents.

**Figure 1:**
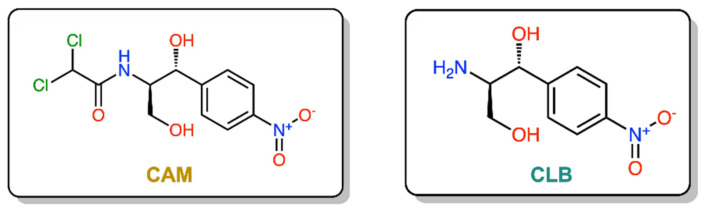
Structures of chloramphenicol (CAM) and chloramphenicol base (CLB).

**Figure 2:**
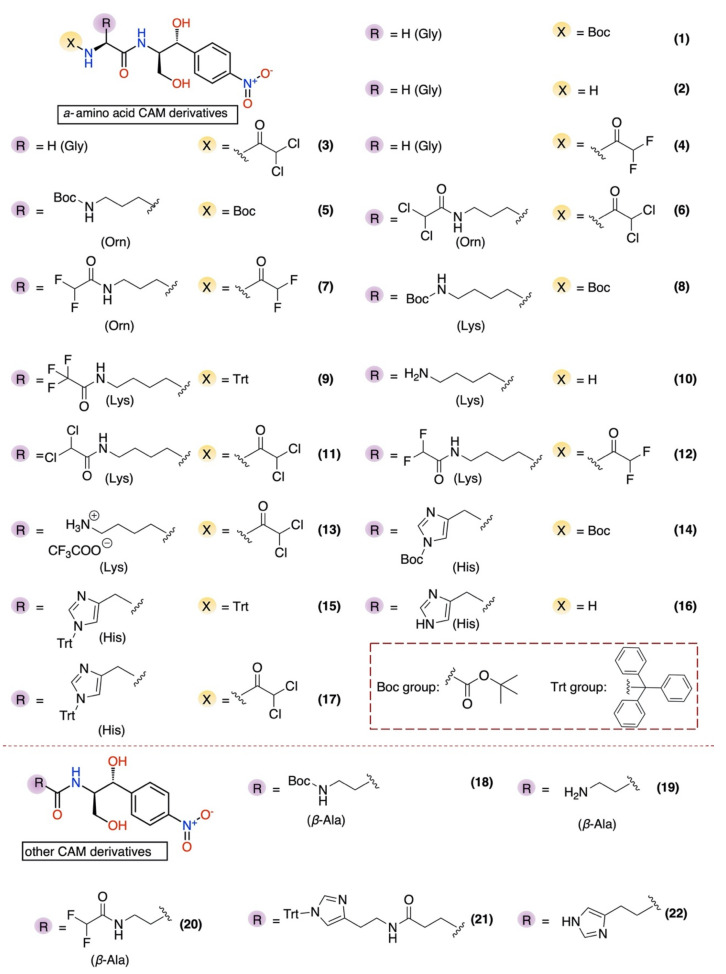
Structures of CLB derivatives encountered in this work.

Discovered in 1947 [5], chloramphenicol (CAM) was the first ribosome-acting and broad-spectrum antibiotic introduced, covering a wide range of gram-positive and gram-negative pathogens. When issued for clinical use, the drug immediately became very popular, not only for its low cost and effectiveness, but also for its few and mild side effects. However, shortly after, its initial mild side effects became serious disorders, like bone marrow depression and aplastic anemia [6,7]. As a result, the toxicity of the drug and the development of safer alternative antibiotics narrowed the indications of chloramphenicol prescriptions and finally its use was declined. Although CAM was downgraded therapeutically, it never ceased to be an attractive tool for protein synthesis studies, especially regarding the ribosome structure and function development. Following numerous functional and structural studies, it has been established that CAM binds in the large ribosomal subunit covering partially the binding surface of the A-site substrate and therefore inhibiting protein synthesis [8,9,10,11]. According to the previous references, the aromatic ring of the ribosome-bound CAM overlaps with the placement of side chains of the incoming aa-tRNAs, thus efficiently preventing the aminoacyl moiety of aa-tRNA from properly accommodating into the peptidyl-transferase center (PTC) active site. This model was recently revised based on the novel data which support the theory that CAM acts as a context-specific inhibitor of translation whose action depends on the nature of specific amino acids in the nascent chain and the identity of the residue entering the A site. More precisely, chloramphenicol-mediated inhibition is stimulated when a nascent peptide in the ribosome carries an alanine amino acid in the penultimate position and preferentially aspartic acid and lysine in the P and A sites, respectively. This ADK (alanine–aspartic acid–lysine) motif, is the most preferable for CAM action and is soundly characteristic of the functional interplay between the nascent chain and the ribosomal peptidyl transferase activity [12,13]. The emergence of undesirable side effects and increasing antimicrobial resistance led to an early onset of studies for chloramphenicol derivatization targeting both characteristics, improved antimicrobial activity and lowered toxicity side effects. Two of the most frequently and rapidly spreading resistance mechanisms operate through the enzymatic modification of either the CAM molecule or the ribosome-binding site. In the first case, CAM is modified via its acetylation with acetyltransferase enzymes (CATs) and in the second, via methylation of the A2503 base of 23S rRNA, with lost affinity in both cases of CAM for the ribosome [14]. To this day, numerous derivatives have been designed and synthesized, but none of them has been evaluated as superior to the parental chloramphenicol molecule (reviewed in [15,16,17,18,19,20]). Although the derivatization approach has not been successful up till now, efforts have never ceased since it is one of the most productive methods for the development of new-generation antibiotics against pathogenic resistance. In this study, using the chloramphenicol base (CLB) as a starting material (Figure 1), we tethered its amine group to the Boc- or Trt-protected amino acids glycine, histidine, lysine, ornithine or β-alanine through an amide bond. These compounds were then deprotected and the thus obtained free amine groups of the amino acid part were further modified, introducing either a dichloroacetic or a difluoroacetic group (Figure 2).

According to the in vivo and in vitro evaluation of all new compounds, the bis-dichloroacetyl derivative of ornithine (6) (Figure 2) displayed the highest antimicrobial activity in vivo and in vitro and appears to be a dynamic new pharmacophore with room for further modification and development.

## 2. Results and Discussion

### 2.1. Chemical Synthesis

For the purpose of this work, we prepared 22 derivatives (33 compounds in total, including intermediates), starting from the commercially available CLB. Detailed synthetic schemes and procedures describing the preparation and the structural characterization by ^1^H, ^13^C NMR and ESI–MS spectra of each of the abovementioned compounds can be found in the Supplementary Materials section of this paper.

### 2.2. Antibacterial Activity

**Figure 3:**
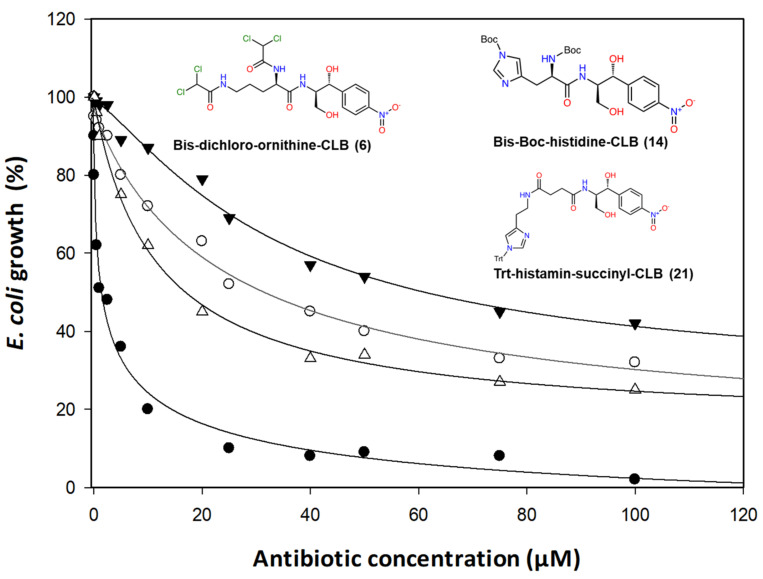
Bacteria *E. coli* ΔTolC growth in the presence of CLB derivatives. The bacterial cultures were grown in the presence of either CAM or each derivative at increasing concentrations (0.05 μΜ up to 100 μM). (•) Chloramphenicol, (Δ) bis-dichloro-ornithine–CLB (compound 6), (○) Trt-histamine–succinyl–CLB (compound 21), (▼) bis-Boc-histidine–CLB (compound 14).

All the synthesized compounds were first tested in vivo. Using an antibiotic-sensitive *Escherichia coli* ΔTolC strain, we monitored culture growth in the presence of increasing concentrations of all antibiotics. Among the compounds tested, only three of them displayed significant inhibition (Figure 3). Expressing the data as percent inhibition of the control grown in the absence of antibiotics, we calculated the EC_50_ concentrations which are presented in Table 1 in both μM and μg/mL, ranging from 6.70 to 18.66 μg/mL (Table 1). Compound 6, which is the most effective, is a derivative of the CLB with the basic amino acid ornithine, where both of its amine groups bear a dichloroacetyl group in the form of an amide bond. The remaining two compounds, 21 and 14, with noticeable in vivo activity are both histidine derivatives, where the amine groups are protected either with Trt as in the case of (21) or with Boc in the case of (14). In reality, compound 21 is not a direct histidine derivative but rather a histamine one, which is tethered to the CLB through a succinic acid linker. Histidine–CLB is well-known from a previous publication [19] for acting as a potent inhibitor of ribosome function, as well as lysine–CLB, and according to their crystal structure analysis bound on the ribosome, the amphenicol part of their molecules occupies the same site as CAM, while the additional part is directed towards the growing peptide chain, namely, the upper place of the tunnel [19].

Next, we tested all the compounds in vitro. First, we tested their effect on overall protein synthesis, using a cell-free transcription–translation system using the *Renilla reniformis* luciferase gene as a template [21].

**Figure 4:**
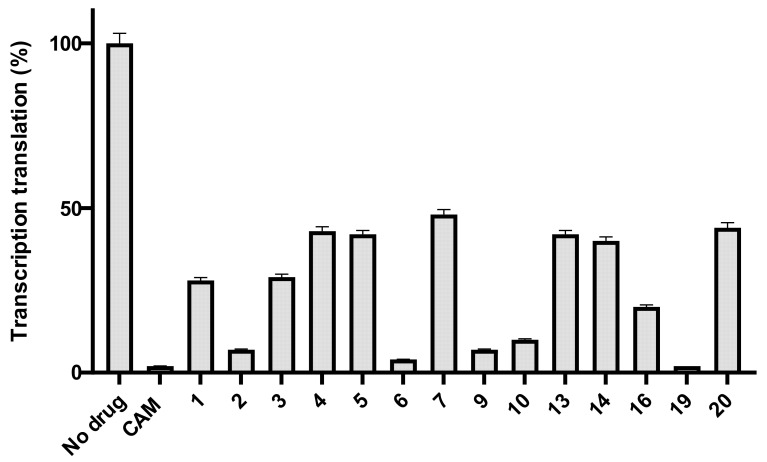
In vitro Transcription–translation system in the presence of CLB derivatives. All compounds of Figure 2 were tested in 20 μM concentration and the most active of them are presented. Products are presented as the percentage of control (without inhibitors). The chemical structure of compounds 2, 6, 9, 10 and 19 is presented in Table 2.

As we can see in Figure 4, many compounds exhibited noticeable inhibition, albeit lower than the molecule of chloramphenicol. Compounds 6 and 9 were the most powerful inhibitors among all the tested compounds and it is important to underline that out of all of them, compound 6 was the most active in vivo as well (Figure 3). Additionally, compounds 1, 2, 3, 4, which are all glycine derivatives, exhibited in vitro inhibition confirming previous publications [19,22], according to which glycine–CLB keeps high affinity for ribosome binding and inhibits its function in vitro. The appearance of the Boc-protective group in compound 1 and the dichloroacetyl or difluoroacetyl group in (3) and (4), respectively, did not have any impact, supporting the notion that inhibition occurs due to the glycine molecule tethered to the CLB rather than to the modified NH_2_. Next, we found compounds 9 and 6 to be equally effective (Figure 4), with the former being a lysine–CLB derivative, for which it is known from previous publications that lysine–CLB converts CLB in a dynamic inhibitor of ribosome function covering also the chloramphenicol binding site [19]. The fact that lysine amino groups are modified in a different way in compounds 9 and 13 and only (9) behaves as a potent inhibitor means that the nature of amine modification is very important. Moreover, the acetylation of amine groups in lysine–CLB either with dichloro- or difluoroacetyl groups (compounds 11 and 12, respectively) abolished the inhibitory activity, while the protection of the ε-amine with trifluoroacetyl and of the α-amine with the Trt group kept the inhibitory activity high. It is also very interesting to compare compounds 6 and 11. The two molecules are very similar, the only difference being the length of the side chain carbon skeleton. In (11), lysine has a methylene group more comparable to ornithine in (6). Interestingly, only the ornithine derivative behaved as an inhibitor either in vivo or in vitro.

In Figure 4, we can also see also a decrease in histidine–CLB activity after NH_2_ modification. As mentioned before, it is known from a previous publication that histidine tethered to the CLB recovers its lost antimicrobial activity in vitro [19]. New protections of free amine groups with a trityl group in compound 15 abolished the inhibition effect (not included in the figure), while in the case of the tert-butyloxycarbonyl group-protected compound 14, it severely decreased the inhibition activity compared to histidine–CLB (18).

**Figure 5:**
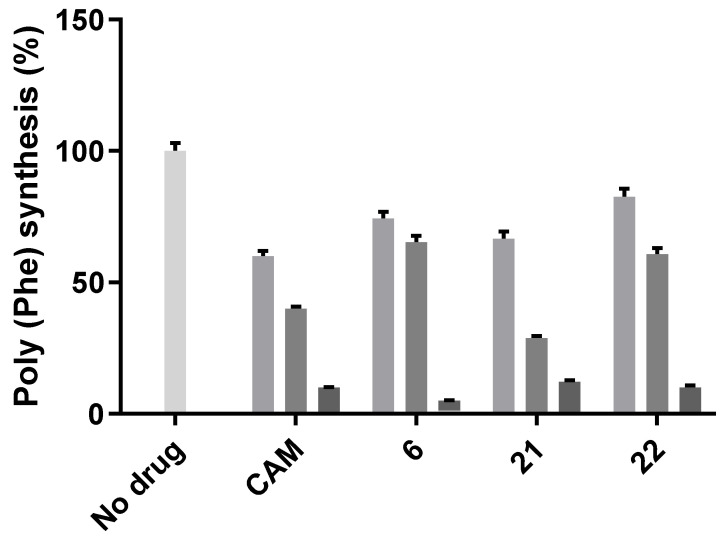
Poly(Phe) synthesis in the presence of CAM and CLB derivatives, at 5, 20 and 50 μM concentrations for each antibiotic, respectively. Results are expressed as percentage of control (without antibiotics).

Next, we tested the polyphenylalanine formation in the presence of antibiotics and the results are presented in Figure 5. Only three compounds exhibited serious inhibition of phenylalanine polymerization, namely, compounds 6, 21 and 22. Compound 6 was also shown to be a powerful inhibitor in vivo (Figure 3), in the transcription–translation assay (Figure 4) and with the inhibition of the poly(Phe) synthesis. Therefore, it was shown to act as a powerful inhibitor of bacterial growth and have a significant impact on the ribosome and peptide bond formation process. Compound 21 is also an antimicrobial agent (Figure 3) inhibiting protein biosynthesis as well, while compound 22 is a powerful inhibitor of polyphenylalanine synthesis, but without any effect on cell growth or in the transcription–translation system. More importantly, this last compound is a dihydro urocanic acid derivative of the CLB which contains the imidazole ring, a basic constituent of the histidine amino acid that is known to participate in histidine–CLB anchoring and inhibition [19]. The incompatibility between the two different in vitro systems is not a surprise as it is known from literature that poly(Phe) is an artifact polymerization system without natural initiation, termination or nascent chain pass steps [23], while the transcription–translation system depends on the mRNA that is translated and/or the type of inhibitor used [24]. Although the last system is as close as possible to a proper representative of in vivo conditions, it has been established in the recent years that inhibitors acting in the peptidyl transferase center are not general inhibitors, but are context-specific, depending on the amino acids, the tRNAs and the translated mRNA sequences [13,14]. Taking into account that CAM is such an inhibitor, it is expected that CAM derivatives could also behave in a context-specific manner.

**Figure 6:**
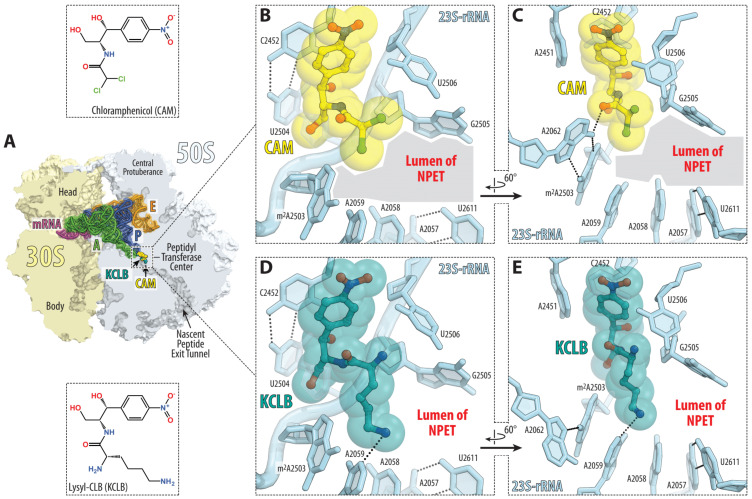
Comparison of the structures of ribosome-bound chloramphenicol and its lysyl derivative. (**A**) Overview of the chloramphenicol (CAM)-binding site (yellow) in a *T. thermophilus* 70S ribosome viewed as a cross-cut section through the ribosome. The 30S subunit is shown in light yellow, the 50S subunit is in light blue, the mRNA is in magenta and the A, P and E site tRNAs are colored green, dark blue and orange, respectively. (**B**–**E**) Close-up views of the CAM (yellow; PDB entry 6ND5) [11] and its lysyl derivative (lysine-chloramphenicol base, KCLB, teal; PDB entry 6CFL) [19] bound in the PTC of the 70S ribosome. The *E. coli* nucleotide numbering is used. H-bond interactions are indicated with dashed lines. Note that either the dichloroacetyl moiety of CAM or the amino acid moiety of KCLB point towards the large open space in the lumen of the nascent peptide exit tunnel (NPET). The extended tail of bis-dichloroacetyl-ornithine–CLB is likely to be accommodated in this space by forming compound-specific interactions with the nucleotides lining up the NPET.

In Figure 6, we can see the known structures of CAM [11] and lysine–CLB bound on the *Thermus thermophilus* 70S ribosomes [19], and it can easily be concluded that there is plenty of space in the lumen of the ribosomal exit tunnel to allow accommodation of bis-dichloroacetyl-ornithine–CLB (6), on which our interest is focused. The potential for binding forces between the tail of the compound and the bases around is obvious, but it depends on the specific accommodation of the molecule, which remains to be examined as the next step. At first glance, potential secondary bonds could be formed between the molecule tail and the bases around, namely, m^2^A503, A2059, A2058, A2057, U2611 and G2505.

From the data presented above, we can conclude that:

1. Basic amino acids ornithine, histidine and lysine as well as glycine but not β-alanine recover the inhibitory activity lost after removal of the dichloroacetyl tail of the chloramphenicol molecule.
2. The imidazole ring is also important as we can see from histidine replacement with dihydro-urocanic acid or in the case of Trt-histamine–succinyl–CLB, compounds 22 and 21, respectively.
3. Dichloroacetylation of free amino groups really helps in the case of ornithine, but not in the case of lysine or histidine.
4. The length of the side chain carbon skeleton of an amino acid is very important for binding and accommodation as we can see comparing dichloroacetyl-ornithine- or lysine–CLB.
5. Protected versus free amine groups assist in recovering the compound’s antimicrobial activity, but not for all cases. For each individual case, it is important to monitor the surrounding interactions and their correlation with the effect to binding forces and inhibitory activity.

In conclusion, the bis-dichloroacetyl-ornithine-CLB derivative 6 displayed the highest antimicrobial activity both in vivo and in vitro and seems to be a new dynamic pharmacophore with room for further modifications and development and our respective experiments will follow; keeping this part of the molecule, we plan to modify the primary and/or secondary hydroxyl groups to improve our molecule characteristics.

## 3. Materials and Methods

### 3.1. Materials

Filter count scintillation liquid was purchased from Perkin Elmer (Richmond, CA, USA), l-[2,3,4,5,6-[^3^H]-phenylalanine was purchased from Amersham Pharmacia Biotech (Piscataway, NJ, USA), while tRNA bulk, ATP and poly(U) were from Merck (Kenilworth, NJ, USA).

### 3.2. Bacterial Strains

All the strains were grown at 37 °C in a Luria-Bertani (LB) medium with continuous shaking. The wildtype *E. coli* K12/MG1655 strain was used to isolate the components of the functional assays such as ribosomes and tRNA. SQ110 strains contain ptRNA67 for tRNA supplementation providing spectinomycin resistance (50 μg/mL). For the ΔTolC strain, we also included 50 μg/mL kanamycin.

### 3.3. Biochemical Preperations

As described by Blaha et al. [25], we prepared 70S re-associated ribosomes from *E. coli* K-12 cells and kept them in a buffer containing 20 mM HEPES/KOH, pH 7.6, 50 mM CH_3_COONH_4_, 6 mM (CH_3_COO)_2_Mg and 4 mM mercaptoethanol. The S-100 fraction was prepared as described by Rheinberger et al. [26] and was treated with DE-52 cellulose to absorb away the tRNAs and most RNases.

### 3.4. Inhibition of Translation Using an E. coli-Based In Vitro Cell-Free Expression System

In this study, we used a S30 T7 high-yield protein expression system (Promega Corporation). We performed screening assays of our compounds in small-scale in vitro transcription–translation reactions using the included control DNA template containing the *Renilla reniformis* luciferase gene as previously described [21]. From each reaction, 2.5 μL samples were taken and diluted by adding 97.5 μL of the lysis buffer from the Renilla Luciferase Assay kit (Biotium) used. The mix was thoroughly mixed, then 50 μL were placed into a 96-well white opaque plate (Greiner). Right before the measurement, 50 μL of the assay reagent were added to all the samples, mixed and immediately placed in a luminometer (Perkin Elmer Victor2) for measurement.

### 3.5. EC_50_ Determination

The cells were grown overnight in a Luria–Bertani medium and were diluted into fresh Luria–Bertani medium again and were grown. Exponential-phase cultures were then diluted to a final *A*_600_ of 0.050 and incubated with each one of the antibiotics at indicated concentrations and 37 °C for the appropriate time to reach absorbance equal to 0.700 of the control sample which was grown without antibiotics.

From dose-response curves, the half-maximal effective concentration (EC_50_) for each compound and strain was estimated. EC_50_ represents the molar concentration of a compound that produces 50% of the maximal possible effect. The EC_50_ values were mathematically determined by non-linear regression fitting of the observed culture’s optical density values expressed as percentage of 0.700 (*y*), into the Hill equation:

(1) $$y=min+\frac{max-min}{1+{\left(\frac{x}{EC50}\right)}^{-n}}$$

where *min* and *max* are the lowest and highest observed values of the culture’s optical density, respectively, *x* is the concentration of the tested compound, and *n* is the Hill coefficient that represents the largest absolute value of the curve slope. EC_50_ is equal to the *x* value of the sigmoid’s midpoint. Fitting was performed using the four parameter logistic curve of the Sigma Plot Program Version 11.0 (Systat Software, Inc., San Jose, CA 95131 USA) for exact graphs and data analysis.

### 3.6. Poly(U)-Dependent Poly(Phe) Synthesis

The assay was carried out in buffer A with re-associated 70S ribosomes and was performed in reaction volumes of 15 μL. Each incubation mixture contained 70S ribosomes (0.20 μM) preincubated with each antibiotic for 10 min at 37 °C, fractionated poly(U) (25 μg), [^3^H]phenylalanine (15 μM, 50 dpm/pmol), bulk tRNA from *E. coli* (1 *A*260 unit), ATP (3 mM), GTP (1.5 mM), acetyl phosphate (5 mM) and S-100 fraction [25]. After 60-min incubation at 37 °C, hot trichloroacetic acid precipitation followed, and polypeptides were isolated on glass fiber filters [27]. The remaining radioactivity on the filters was measured in a liquid scintillation counter.

Figure 6 showing atomic models was generated using the PyMol 2.4 software (update 2021; www.pymol.org accessed on 5 April 2021).

## Figures and Tables

**Table 1:** EC_50_ determination of the most effective derivatives with the *E. coli* ΔtolC strain.

Antibiotic	μM	μg/mL
Chloramphenicol	2.36	0.76
Bis-dichloroacetyl-ornithine	12.25	6.70
Histamine–succinyl–CLB	25.02	13.71
Bis-Boc-histidine–CLB	34.00	18.66

**Table 2:** Chemical structure for the most active compounds of Figure 4.

Compound	Structure
2	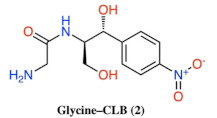
6	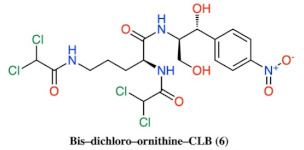
9	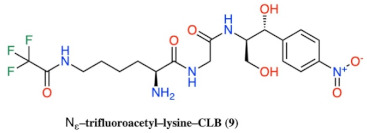
10	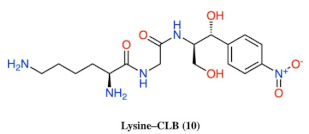
19	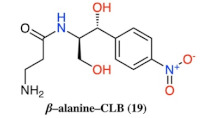

## Data Availability

Not applicable.

## Supplementary Materials

The following are available online at https://www.mdpi.com/article/10.3390/antibiotics10040394/s1, Scheme S1: Synthesis of analogs 1–8, 10–12, 14 and 16 from commercially available Boc-protected amino acids; Scheme S2: Synthesis of analogs 9 and 13 from commercially available *Nε*-trifluoroacetyl-L-lysine; Scheme S3: Synthesis of analogs 15 and 17 from commercially available L-histidine; Scheme S4: Synthesis of analogs 18, 19 and 20 from commercially available Boc-β-alanine; Scheme S5: Synthesis of analog 21 from commercially available histamine; Scheme S6: Synthesis of analog 22 from commercially available urocanic acid. Moreover, the section contains detailed synthetic procedures describing the preparation and the structural characterization by ^1^H, ^13^C NMR and ESI–MS spectra of each compound synthesized for the purposes of this work.
