# Supplementary material for: New Chloramphenicol Derivatives with a Modified Dichloroacetyl Tail as Potential Antimicrobial Agents
Source: Antibiotics (Basel). 2021 Apr 6;10(4):394. doi: 10.3390/antibiotics10040394 (PMC8067500; doi:10.3390/antibiotics10040394)
Supplement: Supplementary file 1 [file antibiotics-10-00394-s001.pdf]

SUPPORTING INFORMATION

Table of Contents

General **1**

1. Experimental **1**

A. Synthesis of analogues 1-8, 10-12, 14 and 16 .... **1**

Synthesis of amides 1, 5, 8, and 14 1

N-Boc deprotection of compounds 1, 5, 8, and 14 2

Synthesis of amides 3, 4, 6, 7, 11 & 12 3

B. Synthesis of analogues 9 and 13 .... **5**

N_α protection of N_ε-Trifluoroacetyl-*L*-lysine with Trityl-group 5

Synthesis of (5-(2,2,2-trifloroacetyl))-2-tritylamino-Lysine-CLB (9) 6

N_ε deprotection of compound 9 6

N_α deprotection of compound 31 7

Synthesis of N_ε-Boc-N_α-dichloroacetyl-Lysine-CLB (33) 7

N-Boc deprotection of compound 33 7

C. Synthesis of analogues 15 and 17 .... **7**

N_α, N_{im}-bis-trityl protection of Histidine 8

Synthesis of N_α, N_{im}-bis-trityl-Histidine-CLB (15) 8

N_α deprotection of compound 15 8

Synthesis of N_α-(2,2-dichloroacetyl)-N_{im}-trityl-Histidine-CLB (17) 9

D. Synthesis of analogues 18, 19 and 20 .... **9**

Synthesis of *N*-Boc-β-Alanine-CLB (18) 9

N-Boc deprotection of compound 18 10

Synthesis of *N*-(2,2-difluoroacetyl)-*β*-Alanine-CLB (20) 10

E. Synthesis of analogue 21 .... **10**

Di-Trityl-protection of Histamine 10

Selective Trt-deprotection of the primary amine of compound 39 11

Synthesis of N_{im}-Trt-Histamine-CLB (21) 11

F. Synthesis of Urocanic-CLB (22) **11**

Catalytic hydrogenation of urocanic acid 12

Synthesis of analogue 22 12

G. References **12**

General

All solvents were dried and/or purified according to standard procedures prior to use. Anhydrous Na_2SO_4 was used for drying solutions and the solvents were then routinely removed at ca. 40 °C under reduced pressure using a rotary vacuum evaporator. All reagents employed in the present work were commercially available and used without further purification. ^1H NMR spectra were obtained at 600.13 MHz and ^{13}C NMR spectra at 150.90 MHz on a Bruker AVANCEIII HD spectrometer. Chemical shifts (δ) are indicated in parts per million (ppm) upfield from TMS and coupling constants (J) are reported in hertz. ESI+ mass spectra were recorded at 30V, on a Micromass-Platform LC spectrometer using MeOH as solvent.

Melting points were determined with a Buchi SMP-20 apparatus and are uncorrected. When required, reactions were carried out under an inert argon atmosphere in preflamed glassware. Flash column chromatography (FCC) was performed on Merck silica gel 60 (230-400 mesh) and analytical thin layer chromatography (TLC) on Merck or Macherey silica gel $60F_{254}$ pre-coated aluminum foils (0.2 mm film). Spots on TLC plates were visualized with UV light at 254 nm and ninhydrine solution.

1. Experimental

A. Synthesis of analogues 1-8, 10-12, 14 and 16

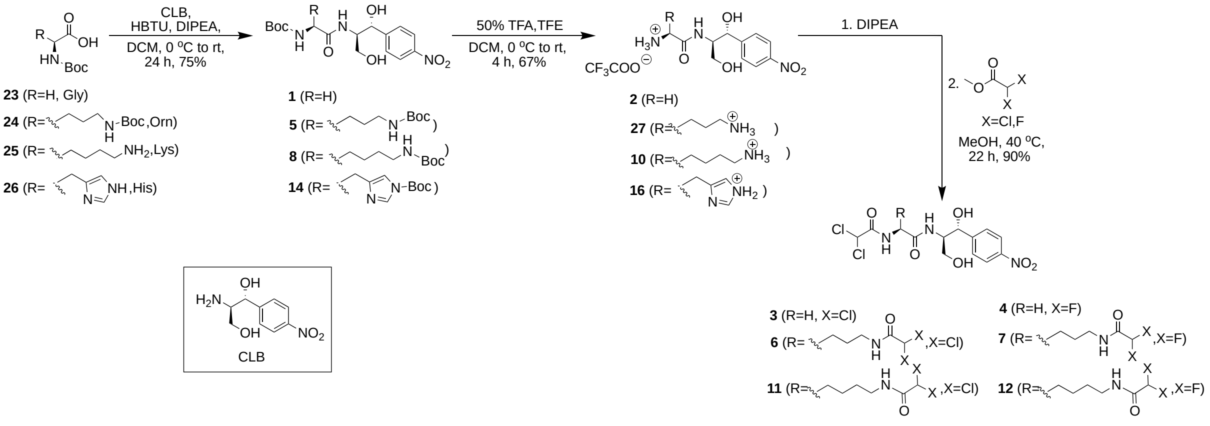

Scheme 1. Synthesis of analogues 1-8, 10-12, 14 and 16 from commercially available Boc-protected amino acids.

Synthesis of amides 1, 5, 8, and 14.[1,2]

To an ice-cold solution of the commercially available Boc-protected amino acid (1.0 eq) in DCM (0.18 M), DIPEA (1.1 eq), HBTU (1.1 eq) and CLB (1.7 eq) was added. The reaction mixture was stirred at room temperature and monitored by TLC. Then, the mixture was evaporated to dryness under vacuum and the residue thus obtained diluted with AcOEt and washed with 5% aqueous $NaHCO_3$, H_2O and brine. The organic layer was dried over Na_2SO_4, filtered and evaporated to dryness under vacuum. The residue was subjected to FCC to give the pure product as a pale yellow oil (75%).

Boc-Glycine-CLB (1)

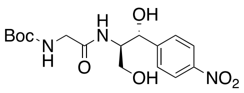

1

R_f (EtOAc, Macherey): 0.16; **^{1}H-NMR** ($CDCl_3$, 600 MHz): δ 8.18 (d, 2H, J = 8.6 Hz), 7.55 (d, 2H, J = 8.6 Hz), 7.07 (br s, 1H), 5.22 (s, 1H), 4.15 (t, 1H, J = 5.9 Hz), 3.99 (unresolved dd, 2H), 3.87 (unresolved dd, 1H), 3.35 (t, 1H, J = 7.8 Hz), 2.88 – 2.83 (m, 1H), 1.97 (br s,

1H), 1.78 (br s, 1H), 1.48 (s, 9H); **^{13}C-NMR** ($CDCl_3$,151 MHz) δ 170.2, 150.0, 148.1, 147.5, 126.8, 123.6, 81.1, 73.8, 63.9, 55.9, 38.6, 28.3; **ESI-MS** (30eV) m/z: 392.36 $[M+Na]^+$.

Di-Boc-Ornithine -CLB (5)

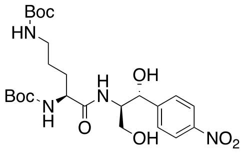

5

R_f (PhMe/EtOAc 4:6, Macherey): 0.12; **^{1}H-NMR** ($CDCl_3$, 600 MHz) δ 8.19 (d, 2H, *J* = 8.4 Hz), 7.58 (d, 2H, *J* = 8.4 Hz), 6.83 (d, 1H, *J* = 8.5 Hz), 5.30 (s, 1H), 5.22 (d, 1H, *J* = 3.5 Hz), 5.13 (unresolved dd, 1H), 4.74 (t, 1H, *J* = 5.8 Hz), 4.11 – 4.04 (m, 2H), 3.94 (dd, 1H, *J* = 11.4, 3.1 Hz), 3.85 (dd, 1H, *J* = 11.5, 3.5 Hz), 3.49 (s, 1H), 3.23 – 3.15 (m, 1H), 2.98 – 2.91 (m, 1H), 1.78 – 1.71 (m, 2H), 1.57 – 1.47 (m, 2H), 1.42 (s, 18H); **^{13}C-NMR** ($CDCl_3$,151 MHz,) δ 184.5, 155.8, 148.4, 147.4, 126.9, 123.5, 81.5, 79.9, 73.9, 63.8, 55.9, 53.9, 41.2, 40.7, 28.4, 28.2, 25.9**;** **ESI-MS** (30eV) m/z: 549.35 $[M+Na]^+$

Di-Boc-Lysine -CLB (8)

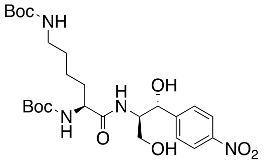

8

R_f (PhMe/EtOAc 1:9, Macherey): 0.2; **^{1}H-NMR** ($CDCl_3$, 600 MHz) δ 8.17 (d, 2H, *J* = 8.3 Hz), 7.57 (d, 2H, *J* = 8.4 Hz), 6.89 (br s, 1H), 5.44 (s, 1H), 5.22 (s, 1H), 4.74 (s, 1H), 4.09 (s, 1H), 3.93 (br s, 2H), 3.81 (unresolved dd, 1H), 3.13 – 2.96 (m, 3H), 2.89 (s, 1H), 1.71 – 1.61 (m, 1H), 1.59 – 1.49 (m, 2H), 1.41 (s, 18H); **^{13}C-NMR** ($CDCl_3$,151 MHz) δ 172.8, 156.8, 156.7, 148.6, 147.3, 126.9, 123.5, 80.6, 79.6, 73.5, 63.6, 55.9, 55.2, 39.3, 38.6, 29.7, 28.4, 28.3, 21.9; **ESI-MS** (30eV) m/z: 563.48 $[M+Na]^+$

Di-Boc-Histidine -CLB (14)

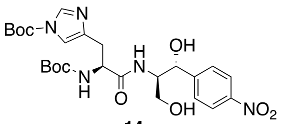

14

R_f (DCM/MeOH 9:1, Macherey): 0.18; **^{1}H-NMR** ($CDCl_3$, 600 MHz) δ 8.2 (d, 2H, *J* = 8.76 Hz), 7.98 (s, 1H), 7.58 (d, 2H, *J* = 8.46 Hz), 7.18 (s, 1H), 6.61 (d, 1H, *J* = 7.38 Hz), 5.56 (s,1H) 5.28 (s, 1H), 5.09 (s, 1H), 4.33 (s, 1H), 4.10 (d, 1H, *J* = 11.16 Hz), 3.92 (s, 1H), 3.80 (d, 1H, *J* = 11.52 Hz), 3.14 – 2.96 (m, 1H), 1.61 (s, 9H), 1.42 (s, 9H); **^{13}C-NMR** ($CDCl_3$,151 MHz) δ 170.8, 148.1, 147.4, 146.4, 137.0, 129.0, 128.2, 127.0, 126.7, 125.3, 123.6, 123.4, 115.8, 86.3, 74.0, 62.9, 56.6, 53.4, 31.1, 29.7, 28.3, 27.8; **ESI-MS** (30eV) m/z: 572.42 $[M+Na]^+$

N-Boc deprotection of compounds 1, 5, 8, and 14

To an ice-cold solution of Boc-protected amine (1.0 eq) in DCM (0.6 M), a solution of 50% TFA (3.0 eq) in DCM and TFE (2.0 eq) were added. The reaction mixture was stirred for 30 min at 0 ^{o}C and then for 3.5 h at room temperature. Upon completion of the reaction the mixture was triturated with Et_2O and Hex to give the desirable product, as a yellow oil in 67% yield. The deprotected intermediate was used without further purification in the next step.

TFA salt of Glycine-CLB (2)

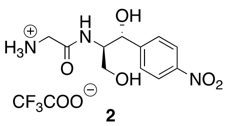

2

R_f (DCM/MeOH/NH_3 9:1:0.1, Macherey): 0.1

Bis-TFA salt of Ornithine-CLB (27)

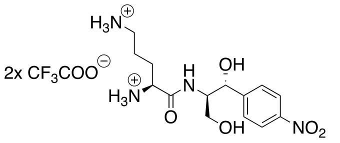

27

R_f (DCM/MeOH/NH_3 8:2:0.2, Macherey): 0.07

Bis-TFA salt of Lysine-CLB (10)

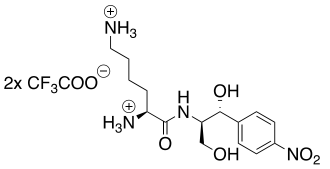

10

R_f (DCM/MeOH/NH_3 8:2:0.2, Macherey): 0.1

Bis-TFA salt of Histidine-CLB (16)

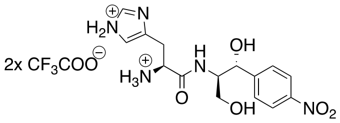

16

R_f (DCM/MeOH/NH_3 8:2:0.2, Macherey): 0.2

Synthesis of amides 3, 4, 6, 7, 11 & 12

To an ice-cold solution of the deprotected intermediates **2**,**10**,**16**,**27** (1.0 eq) in dry MeOH (0.25 M), DIPEA (1.2 eq) was added and the reaction mixture was stirred at 0 ^{o}C for 30 min and then at room temperature. After 10 min methyl dichloroacetate or methyl difluoroacetate was added dropwise (3 eq in case of amides **3** & **4** and 6 eq in case of amides **6**, **7**,**11** and **12** and the reaction mixture was heated at 40 ^{o}C overnight. The following day the mixture was evaporated to dryness under reduced pressure and the residue thus obtained was subjected to FCC to afford the pure conjugates **3**, **4**, **6**, **7**, **11** and **12**.[3]

(2,2-dichloroacetyl)-Glycine-CLB (3)

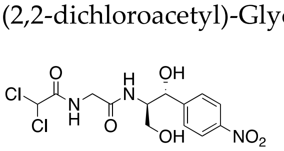

3

R_f (EtOAc, Macherey): 0.16; **^{1}H-NMR** (DMSO-d$_6$, 600 MHz) δ 7.97 (t, 1H, J = 6.1 Hz), 7.31 (d, 2H, J = 8.9 Hz), 6.89 (d, 1H, J = 9.2 Hz), 6.75 (d, 2H, J =8.3 Hz), 5.03 (d, 1H, J = 4.7 Hz), 4.18 (unresolved t, 1H), 4.03 (t, 1H , J = 4.7 Hz), 3.15 – 3.10 (m, 1H), 2.93 (dd, 1H, J = 16.5, 5.9 Hz), 2.81 (dd, 1H, J = 16.5, 5.7 Hz), 2.74 – 2.68 (m, 1H), 1.67 (quintet, 2H, J =1.9 Hz); **^{13}C-NMR** (DMSO-d$_6$, 151 MHz) δ 167.9, 152.2, 146.8, 127.8, 123.3, 108.7, 103.9, 69.6, 60.8, 56.6, 41.8; **ESI-MS** (30eV) m/z: 402.25 [M+Na]$^+$

(2,2-difluoroacetyl)-Glycine-CLB (4)

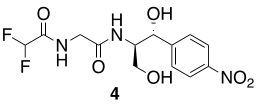

4

R_f (EtOAc, Macherey): 0.12; **^{1}H-NMR** (DMSO-d_6, 600 MHz) δ 8.56 (t, 1H, *J* = 5.6 Hz), 8.15 (d, 2H, *J* = 8.9 Hz), 7.74 (d, 1H , *J* = 9.2 Hz), 7.59 (d, 2H, *J* = 8.8 Hz), 5.84 (d, 1H, *J* = 4.5 Hz), 5.00 (unresolved t, 1H), 4.84 (dd, 1H *J* =6.4, 4.7 Hz), 3.97 – 3.92 (m, 1H), 3.75 (dd, 1H, *J* = 16.7, 5.8 Hz), 3.67 (dd, 1H, *J* = 16.7, 5.5 Hz), 3.58 – 3.54 (m, 1H) 2.48 (m, 2H); **^{13}C-NMR** (DMSO-d_6, 151 MHz) δ 167.9, 164.0, 152.2, 146.8, 129.3, 128.6, 127.8, 123.3, 69.9, 66.9, 60.9, 56.6, 42.7; **ESI-MS** (30eV) m/z: 370.33 [M+Na]$^+$

2. ,5-bis(2,2-dichloroacetyl)-Ornithine-CLB (6)

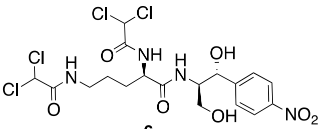

6

R_f (PhMe/EtOAc 1:9, Merck): 0.11; **^{1}H-NMR** (DMSO-d_6, 600 MHz) δ 8.52 (t, 1H, *J* = 5.9Hz), 8.46 (d, 1H, *J* = 8.5 Hz), 8.07 (d, 2H, *J* = 8.8 Hz), 7.91 (d, 1H, *J* = 9.2 Hz), 7.54 (d, 2H, *J* = 8.8 Hz), 6.41 (s, 1H), 6.38 (s, 1H), 5.83 (d, 1H, *J* =4.7 Hz), 5.73 (s, 2H), 5.00 (s, 1H), 4.83 (br s, 1H), 4.33 (q, 1H, *J* =8.1 Hz), 3.95 – 3.89 (m, 1H), 3.56 (t, 1H, *J* = 8.5 Hz), 3.08 – 2.99 (m, 2H), 1.54 – 1.27 (m, 4H); **^{13}C-NMR** (DMSO-d_6, 151 MHz) δ 170.7, 163.9, 163.4, 152.1, 146.8, 127.8, 123.2, 69.7, 67.4, 66.9; **ESI-MS** (30eV) m/z: 569.10 [M+Na]$^+$

2. ,5-bis(2,2-difluoroacetyl)-Ornithine-CLB (7)

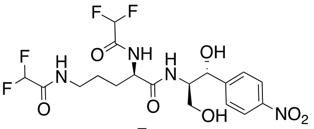

7

R_f (PhMe/EtOAc 1:9, Merck): 0.12, ; **^{1}H-NMR** (DMSO-d_6, 600 MHz) δ 8.74 (d, 2H, *J* = 8.4 Hz), 8.09 (d, 2H, *J* = 8.4 Hz), 7.84 (d, 1H, *J* = 9.2 Hz), 7.56 (d, 2H, *J* = 8.8 Hz), 6.16 (td, 2H, *J* = 4.8 Hz), 5.85 (d, 1H, *J* = 4.5 Hz), 5.76 (s, 1H), 5.03 – 4.99 (m, 1H), 4.83 (t, 1H, *J* = 5.4 Hz), 4.35 (q, 1H, *J* = 8.4 Hz), 3.97 – 3.90 (m, 1H), 3.60 – 3.53 (m, 1H), 3.07 (q, 2H, *J* =6.8 Hz), 1.55 – 1.26 (m, 4H); **^{13}C-NMR** (DMSO-d_6, 151 MHz) δ 170.7, 152.0, 146.7, 127.8, 123.0, 109.2 (q, J_{CF} = 98.6 Hz), 69.6, 60.2, 56.4, 52.4, 38.6, 29.7, 25.4; **ESI-MS** (30eV) m/z: 505.32 [M+Na]$^+$

2. ,6-bis(2,2-dichloroacetyl)-Lysine-CLB (11)

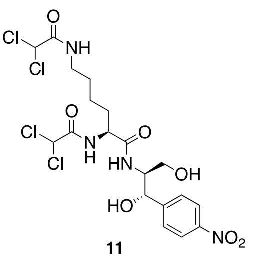

11

R_f (PhMe/EtOAc 1:9, Merck): 0.13; **^{1}H-NMR** (DMSO, 600 MHz) δ 8.50 (t, 1H, *J* = 4.9 Hz), 8.44 (d, 1H, *J* = 8.5 Hz), 8.07 (d, 2H, *J* = 8.6 Hz), 7.91 (d, 2H, *J* = 9.2 Hz), 7.55 (d, 2H, *J* = 8.6 Hz,), 6.41 (d, 2H, *J* = 17.5 Hz,), 5.82 (d, 1H, *J* = 4.5 Hz), 5.75 (s, 1H), 5.02 (br s, 1H), 4.33 – 4.27 (m, 1H), 3.95 (q, 1H, *J* = 6.1 Hz), 3.61 – 3.54 (m, 1H), 3.07 – 3.01 (m, 2H), 1.54 – 1.33 (m, 4H); **^{13}C-NMR** (DMSO-d_6, 151 MHz) δ 170.9, 163.9, 163.4, 152.2, 146.7, 127.8, 123.2, 69.7, 67.4, 67.0, 60.2, 56.3, 55.4, 53.1, 32.3, 28.5, 22.7; **ESI-MS** (30eV) m/z: 583.22 [M+Na]$^+$

2. ,6-bis(2,2-difluoroacetyl)-Lysine-CLB (12)

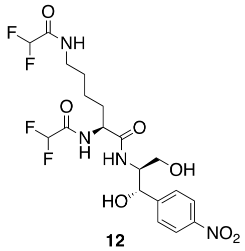

12

R_f (PhMe/EtOAc 1:9, Merck): 0.11; **^{1}H-NMR** (DMSO-d$_6$, 600 MHz) δ 8.71 (d, 2H, *J* = 8.3 Hz), 8.09 (d, 2H, *J* = 8.7 Hz), 7.82 (d, 1H, *J* = 9.2 Hz), 7.54 (d, 2H, *J* = 8.7 Hz), 6.14 (td, 2H, *J* = 1.92 Hz), 5.84 (d, 1H, *J* = 4.5 Hz), 5.75 (s, 1H), 5.03 – 4.98 (m, 1H), 4.83 – 4.79 (m, 1H), 4.33 – 4.27 (m, 1H), 3.96 – 3.91 (m, 1H), 3.60 – 3.54 (m, 1H), 3.05 (q, 2H, *J* = 6.9 Hz), 1.54 – 1.34 (m, 4H); **^{13}C-NMR** (DMSO-d$_6$, 151 MHz) δ 170.9, 162.5, 152.1, 146.8, 127.9, 123.1, 108.0 (q, J_{CF} = 69 Hz), 79.2, 69.8, 60.8, 56.4, 52.8, 38.8, 31.8, 29.4, 28.6, 22.8; **ESI-MS** (30eV) m/z: 519.92 [M+Na]$^+$

B. Synthesis of analogues 9 and 13

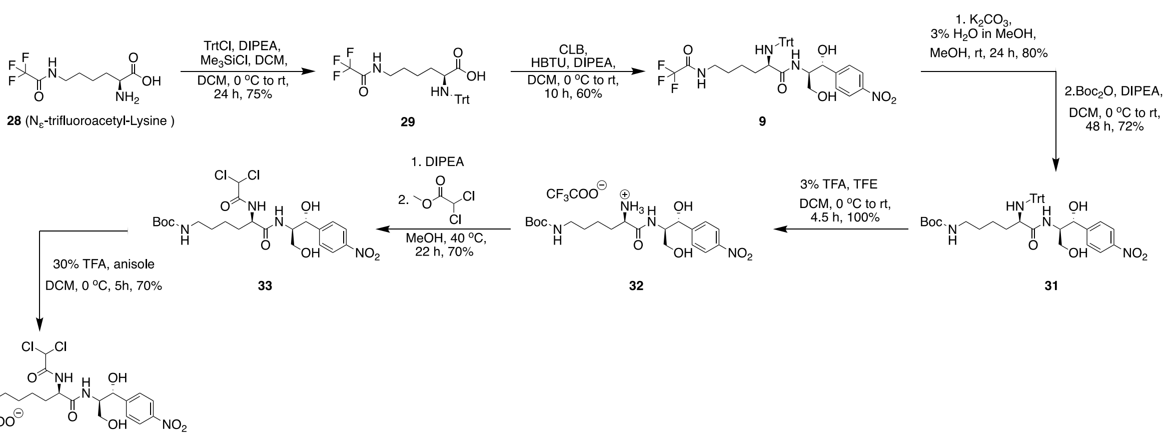

Scheme 2. Synthesis of analogues 9 and 13 from commercially available N$_\varepsilon$-Trifluoroacetyl-L-lysine.

N$_\alpha$ protection of N$_\varepsilon$-Trifluoroacetyl-L-lysine with Trityl-group.[4]

To a solution of the commercially available *N*$_\varepsilon$-Trifluoroacetyl-*L*-lysine (1.0 eq) in DCM (0.33 M), Me$_3$SiCl (1.0 eq) was added and the reaction mixture was stirred at 30 ^{o}C for 2h. Then, it was cooled at 0 ^{o}C and was added DIPEA (2.0 eq) and a solution of TrtCl (1.0 eq) in DCM (0.33 M) dropwise. After 30 min the reaction mixture was allowed to warm up at room temperature and left under stirring overnight. Upon completion of the reaction, MeOH was added at 0 ^{o}C and the mixture was stirred for 30 min. Then, it was evaporated to dryness under reduced pressure and the obtained residue diluted with DCM and washed with 5% aqueous citric acid, H_2O and brine. The organic phase was dried over Na_2SO_4, filtered and evaporated to dryness under vacuum. The residue was subjected to FCC (PhMe/AcOEt 6:4) to give the pure product **29** as a pale yellow oil (75%).

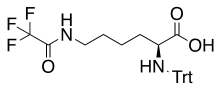

29

R_f (PhMe/EtOAc 6:4, Merck): 0.25; **^{1}H-NMR** (CDCl$_3$, 600 MHz) δ 7.46 – 7.40, 7.32 – 7.24 and 7.23 – 7.14 (3m, 15H), 6.45 (br s, 1H), 3.40 (q, 1H, *J* = 4.5 Hz), 3.31 (q, 2H, J = 6.8 Hz), 2.36 (s, 1H), 1.57 – 1.38 (m, 4H); **^{13}C-NMR** (CDCl$_3$, 151 MHz) δ 177.4, 157.4, 145.2,

129.0, 128.7, 128.1, 127.9, 126.9, 117.4, 71.6, 56.0, 39.5, 33.9, 28.6, 22.0; **ESI-MS** (30eV) m/z: 507.42 $[M+Na]^+$

Synthesis of (5-(2,2,2-trifloroacetyl))-2-tritylamino-Lysine-CLB (9)

To an ice-cold solution of Trt-protected amino acid (1.0 eq) in DCM (0.18 M), DIPEA (1.1 eq.), HBTU (1.1 eq.) and CLB (1.7 eq) were added. The reaction mixture was stirred at room temperature for 10 h and it was monitored by TLC. Then, it was evaporated to dryness under vacuum, diluted with DCM and washed with 5% aqueous citric acid, H_2O and brine. After being dried over Na_2SO_4, the organic extract was filtered and evaporated to dryness under vacuum. The residue was subjected to FCC (PhMe/AcOEt 1:9) to give the pure product **9** as a pale yellow oil (58%).

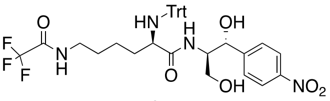

9

R_f (PhMe/EtOAc 1:9, Merck): 0.4; **^{1}H-NMR** ($CDCl_3$, 600 MHz) δ 8.17 (d, 2H, J = 8.6 Hz), 7.86 (d, 1H, J = 8.2 Hz), 7.57 (d, 2H, J = 8.6 Hz), 7.33 – 7.22 (m, 15H), 6.80 (br s, 1H), 5.10 (d, 1H, J = 3.9 Hz), 3.81 (sextet 1H, J = 3.9 Hz), 3.57 (dd, 1H, J = 11.3 & 3.2 Hz), 3.34 – 3.31 (m, 2H), 3.15 (sextet, 1H, J = 6.8 Hz), 3.03 (sextet, 1H, J = 6.4 Hz),), 2.53 (br s, 1H), 1.68 (br s, 1H), 1.47– 1.37 (m, 1H), 1.26 – 1.13 (m, 2H), 1.28 – 1.17 (m, 1H), 1.06 – 0.98 (m, 1H), 0.96 – 0.85 (m, 1H), 0.75 – 0.64 (m, 1H); **^{13}C-NMR** ($CDCl_3$, 151 MHz) δ 175.5, 148.8, 147.4, 145.3, 129.0, 128.1, 127.2, 126.9, 123.4, 74.2, 71.9, 63.9, 57.5, 55.2, 39.0, 34.1, 28.0, 21.2; **ESI-MS** (30eV) m/z: 701.47 $[M+Na]^+$

N_ε deprotection of compound 9

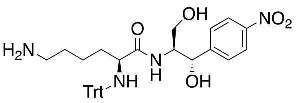

30

R_f (DCM/MeOH 9:1, Merck): 0.16; To a solution of amide 9 (1.0 eq) in MeOH (0.59 M), water (0.07 mL) and K_2CO_3 (12.0 eq) were added. The reaction mixture was stirred at room temperature overnight. Then it was evaporated to dryness under vacuum, diluted with water and washed with DCM. The combined organic phases were washed with brine, dried over Na_2SO_4, filtered and evaporated to dryness under reduced pressure affording the deprotected intermediate 30 with no need for further purification.

N_ε-Boc-protection of amine 30

To an ice-cold solution of amine **30** (1.0 eq) in DCM (0.4 M), DIPEA (1.5 eq), and Boc_2O (1.1 eq) were added. The reaction mixture was stirred at room temperature overnight and was monitored by TLC. Then, the mixture was diluted with DCM and the organic phase was washed with 5% aqueous citric acid, H_2O and brine. After being dried over Na_2SO_4, the organic extract was filtered and evaporated to dryness under vacuum. The residue was subjected to FCC (PhMe/AcOEt 9:1) to give product **31** as a colorless oil (72%).

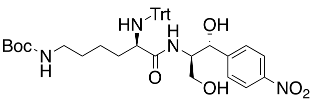

31

R_f (PhMe/EtOAc 9:1, Merck): 0.19; **^{1}H-NMR** ($CDCl_3$, 600 MHz) δ 8.19 (d, 2H, J = 8.8 Hz), 7.76 (d, 1H, J = 8.6 Hz), 7.59 (d, 2H, J = 8.6 Hz), 7.33 – 7.21 (m, 15H), 7.17 (d, 1H, J = 7.0 Hz, 2H), 4.92 (t, 1H, J = 4.7 Hz,), 4.51 (br s, 1H), 4.04 (m, 2H), 3.73 – 3.51 (m, 2H), 3.32 (t, J = 6.1 Hz, 1H), 2.92 – 2.78 (m, 2H), 1.49 (s, 9H), 1.16 – 1.06 (m, 2H), 0.95 – 0.76 (2m, 3H); **^{13}C-**

NMR ($CDCl_3$, 151 MHz) δ 153.7, 148.4, 147.4, 145.3, 129.7, 128.9, 128.1, 127.0, 123.5, 83.2, 72.0, 64.9, 64.1, 57.6, 53.4, 28.4; **ESI-MS** (30eV) m/z: 705.38 $[M+Na]^+$

N_α selective deprotection of compound 31

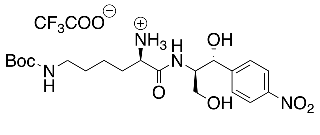

32

R_f (DCM/MeOH 9:1, Merck): 0.34; To an ice-cold solution of **31** (1.0 eq) in DCM (0.26 M), 3% solution of TFA (1.4 eq) in DCM and TFE (1.3 eq) were added. The reaction mixture was stirred at 0°C for 30 min and at room temperature for another 3.5 h. Upon completion of the reaction, the mixture was triturated with Et_2O to give the desirable product **32** as a yellow oil in 100% yield; The deprotected intermediate was used in the next step with no further purification. R_f (DCM/MeOH 9:1, Merck): 0.34

Synthesis of N_ε-Boc-N_α-dichloroacetyl-Lysine-CLB (33)

To an ice-cold solution of the deprotected intermediate **32** (1.0 eq) in dry MeOH (0.25 M) DIPEA (1.25 eq) was added and the mixture was stirred at 0 °C for 30 min and for 10 min at room temperature. Then methyl dichloroacetate (3.0 eq) was added dropwise and the reaction mixture was heated at 40 C overnight. The following day it was evaporated to dryness under vacuum and the residue was subjected to FCC (PhMe/AcOEt 8:2) and affording the pure conjugate **33** as a pale yellow oil in 70% yield.[3]

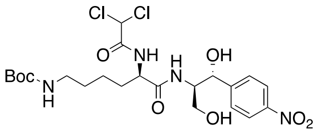

33

R_f (PhMe/EtOAc 8:2, Merck): 0.1; **^{1}H-NMR** ($CDCl_3$, 600 MHz) δ 8.19 (d, 2H, *J* = 8.8 Hz), 7.61 (m, 1H), 7.54 (d, 2H, *J* = 8.5 Hz), 7.32 (d, 1H, *J* = 8.4 Hz), 5.97 (s, 1H), 5.55 – 5.27 (m, 2H), 5.10 (d, 1H, *J* = 2.2 Hz), 4.35 – 4.32 (m, 2H), 4.18 – 4.10 (m, 2H), 3.32 (q, 1H, *J* = 6.5 Hz) 3.08 (s, 2H), 2.82 – 2.78 (m, 2H),), 1.58 – 1.54 (m, 2H), 1.5 (s, 9H); **^{13}C-NMR** ($CDCl_3$,151 MHz) δ 155.6, 137.8, 127.9, 123.6, 82.3, 77.8, 55.1, 51.1, 44.5, 42.1, 29.7, 28.1, 27.7; **ESI-MS** (30eV) m/z: 392.36 $[M+Na]^+$

N-Boc deprotection of compound 33

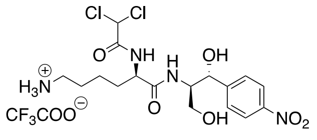

13

R_f (AcOEt, Merck): 0.1; To an ice-cold solution of **33** (1.0 eq) in DCM (0.25 M), a solution of 30% TFA (1.5 eq) in DCM and anisole (1.3 eq) were added and the reaction mixture was stirred at 0 °C for 4 h. Then the mixture was triturated with Et_2O to give the desirable product **13** as a yellow oil in 70% yield; The deprotected intermediate was further treated without any further purification. R_f (AcOEt, Merck): 0.1

C. Synthesis of analogues 15 and 17

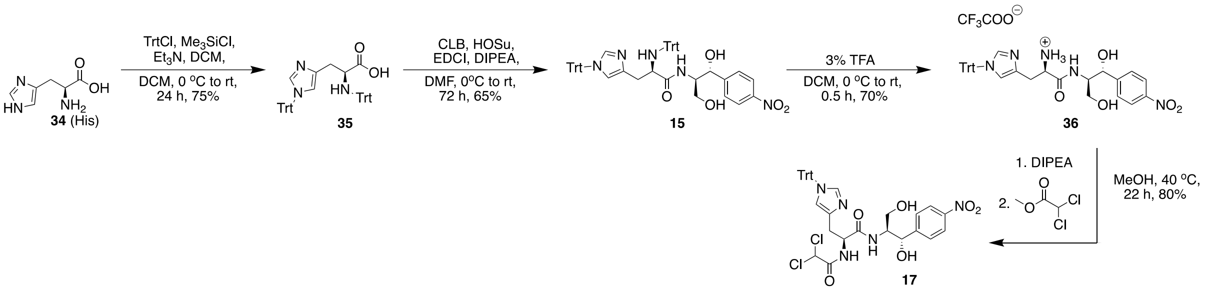

Scheme 3. Synthesis of analogues 15 and 17 from commercially available L-histidine.

N_a , N_{im}-bis-trityl protection of Histidine [4]

To a solution of *L*-histidine (1.0 eq) in DCM (0.33 M), Me_3SiCl (1.0 eq) was added and the reaction mixture was heated at 30°C for 2 h. Then, it was allowed to cool down to room temperature and a solution of Et_3N (1.0 eq) and TrtCl (2.0 eq) in DCM (0.66 M) was added dropwise. After 8 h the reaction mixture was cooled at 0 °C, MeOH was added and left under stirring at 0 °C for 30 min. Then, it was evaporated to dryness under vacuum and diluted with DCM. The organic phase was washed with 5% aqueous citric acid, H_2O and brine. After being dried over Na_2SO_4, the organic extract was filtered and evaporated to dryness under vacuum. The residue was subjected to FCC (PhMe/AcOEt 1:1) to afford pure **35** as a yellow solid (90%).

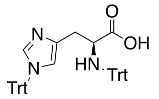

35

R_f (PhMe/EtOAc 1:1, Merck): 0.22; mp 183-185 °C; **^{1}H-NMR** ($CDCl_3$, 600 MHz) δ 7.40 – 7.32 (m, 15H), 7.21 – 7.07 (m, 15H), 6.37 (s, 1H), 3.63 (dd, 1H, J = 7.4, 2.9 Hz), 2.49 – 2.21 (m, 3H), 1.26 (s, 1H); **^{13}C-NMR** ($CDCl_3$,151 MHz) δ 145.5, 129.7, 128.8, 128.4, 128.2, 128.0, 126.6; **ESI-MS** (30eV) m/z: 662.58 $[M+Na]^+$

Synthesis of N_a, N_{im}-bis-trityl-Histidine-CLB (15)

To an ice-cold solution of Trt-protected amino acid **35** (1.0 eq) in DMF (0.5 M), HOSu (1.3 eq) and EDCI (1.3 eq) were added. The reaction mixture was stirred at room temperature overnight. Afterwards DIPEA (1.3 eq.), and CLB (1.1 eq) were added at 0 °C. It was then allowed to gradually warm up to room temperature. After overnight stirring the reaction mixture was diluted with AcOEt and washed with 5% aqueous citric acid, H_2O and brine. The organic phase was dried over Na_2SO_4, filtered and evaporated to dryness under vacuum. The residue was subjected to FCC (PhMe/AcOEt 2:8) to give the pure product **15** as a pale yellow oil (65%).[1,2]

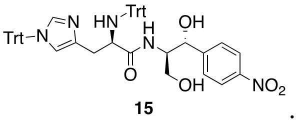

15 .

R_f (PhMe/EtOAc 2:8, Merck): 0.19; **^{1}H-NMR** ($CDCl_3$, 600 MHz) δ 8.23 (d, 2H, J = 8.2 Hz), 7.95 (s, 1H), 7.67 (d, 2H, J = 8.3 Hz), 7.52 (s, 1H), 7.23 – 7. 00 (m, 30H), 6.26 (s, 1H), 5.06 (d, 1H, J = 6.7 Hz), 4.01 (s, 1H), 3.76 (s, 1H), 3.68 (d, 1H, J = 11.5 Hz), 3.53 – 3.45 (m, 1H), 2.99 (d, 1H, J = 11.4 Hz), 2.36 (s, 1H), 1.26 (s, 2H), 0.86 (s, 1H); **^{13}C-NMR** ($CDCl_3$,151 MHz) δ 183.6, 169.6, 160.0, 145.8, 129.5, 128.4, 128.3, 127.9, 127.5, 126.7, 123.6, 73.5, 71.7, 55.5; **ESI-MS** (30eV) m/z: 834.17 $[M+H]^+$, 856.15 $[M+Na]^+$

N_α selective deprotection of compound 15 [4]

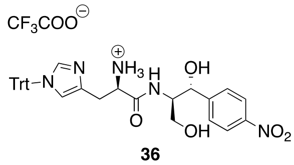

R_f (DCM/MeOH/NH_3 95:5:0.1, Merck): 0.38; To an ice-cold solution of **15** (1.0 eq) in DCM (0.04 M), a solution of 3% TFA (3.0 eq) in DCM and TFE (1.3 eq) were added and the reaction mixture was stirred at 0 °C. After 15 min it was evaporated to dryness under vacuum, diluted with DCM and washed with 5% aqueous $NaHCO_3$, H_2O and brine. The organic phase was dried over Na_2SO_4, filtered and evaporated to dryness under reduced pressure to afford the deprotected intermediate **15**, which was used in the next step without any further purification.[5] R_f (DCM/MeOH/NH_3 95:5:0.1, Merck): 0.38

Synthesis of N_a-(2,2-dichloroacetyl)-N_{im}-trityl-Histidine-CLB (17)

To an ice-cold solution of the deprotected intermediate **15** (1.0 eq) in dry MeOH (0.25 M) DIPEA (1.25 eq) was added and the mixture was stirred at 0 °C for 30 min and for another 15 min at room temperature. Then, methyl dichloroacetate (3.0 eq) was added dropwise and the reaction mixture was heated at 40 C overnight. The following day, it was evaporated to dryness under vacuum and the residue was subjected to FCC (AcOEt) and affording the pure conjugate **17** as a pale yellow oil in 80% yield.[3]

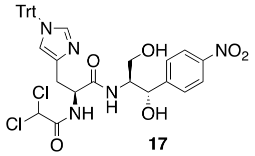

R_f (EtOAc, Merck): 0.2; **^{1}H-NMR** (DMSO-d_6, 600 MHz) δ 8.47 (d, 1H, J = 8.2 Hz), 8.08 (d, 2H, J = 8.8 Hz), 7.83 (d, 1H, J = 9.1 Hz), 7.57 (d, 2H, J = 8.2 Hz), 7.40 – 7.33 (m, 10H), 7.06 (m, 6H), 6.63 (s, 1H), 6.40 (d, 1H, J = 8.8 Hz), 5.79 (d, 1H, J = 4.6 Hz), 5.00 – 4.93 (m, 2H), 4.56 – 4.52 (m, 1H), 3.92 – 3.90 (m, 1H), 3.82 (s, 1H), 2.79 – 2.68 (m, 2H), 1.22 (s, 2H); **^{13}C-NMR** (DMSO-d_6,151 MHz) δ 170.3, 148.2, 142.7, 136.6, 129.7, 128.7, 128.6, 128.5, 128.4, 128.2, 128.0, 127.8, 127.1, 123.2, 81.0, 74.9, 56.7; **ESI-MS** (30eV) m/z: 724.85 [M+Na]$^+$

D. Synthesis of analogues 18, 19 and 20

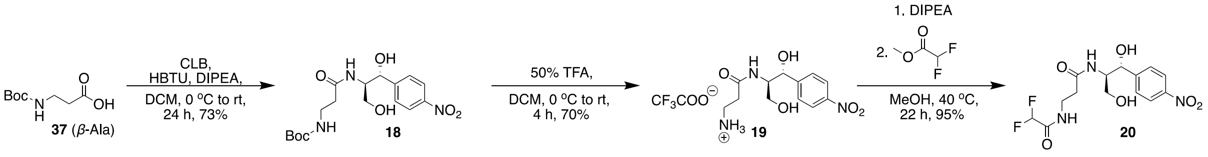

Scheme 4. Synthesis of analogues 18, 19 and 20 from commercially available Boc-β-Alanine.

Synthesis of N-Boc-β-Alanine-CLB (18)

To an ice-cold solution of the commercially available Boc-β-Alanine (1.0 eq) in DCM (0.18.M), DIPEA (1.1 eq.), HBTU (1.1 eq.) and CLB (1.7 eq) were added. The reaction mixture was stirred at room temperature to complete the reaction (monitored by TLC) and then, evaporated to dryness under vacuum. The residue was diluted with AcOEt, washed with 5% aqueous $NaHCO_3$, H_2O and brine, dried over Na_2SO_4, filtered and evaporated again to dryness. The residue thus obtained was subjected to FCC, using EtOAc as eluent, affording the pure product **18** as a pale yellow oil (73%).[1,2]

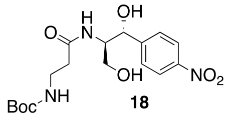

Rf (EtOAc, Merck): 0.1; **1H-NMR** ($CDCl_3$, 600 MHz) δ 8.22 (d, 2H, *J* = 8.7 Hz), 7.57 (d, 2H, *J* = 8.6 Hz), 6.58 (s, 1H), 5.21 (d, 1H, *J* = 3.1 Hz), 4.98 (br s, 1H), 4.13 (br s, 1H), 3.89 (dd, 2H, *J* = 4.2, 11.4 Hz), 3.83 (dd, 1H, *J* = 3.7, 11.3), 3.38 (br s, 1H), 3.30 – 3.25 (m, 1H), 2.80(s, 1H), 2.40 – 2.24 (m, 2H), 1.42 (s, 9H) ppm; **13C-NMR** ($CDCl_3$,151 MHz) δ 172.0, 147.4, 126.8, 123.5, 63.7, 55.9, 38.6, 38.0, 37.1, 28.3; **ESI-MS** (30eV) m/z: 406.51 $[M+Na]^+$

N-Boc deprotection of compound 18

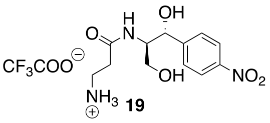

19

Rf (DCM/MeOH/NH_3 9:1:0.1, Macherey): 0.1; To an ice-cold solution of **18** (1.0 eq) in DCM (0.6 M), a solution of 50% TFA (3.0 eq) in DCM and TFE (2.0eq.) were added. After 30 min at 0°C and another 3.5 h at room temperature the mixture was triturated with Et_2O and Hex to give **18** in the form of its trifluoroacetate salt, as a yellow oil in 70% yield, which was used in the next step with no further purification. Rf (DCM/MeOH/NH_3 9:1:0.1, Macherey): 0.1

Synthesis of N-(2,2-difluoroacetyl)-β-Alanine-CLB (20)

To an ice-cold solution of the deprotected intermediate **19** (1.0 eq) in dry MeOH (0.25 M) DIPEA (1.25 eq) was added, and the mixture was stirred at 0 °C for 30 min and for 15 min at room temperature. Then, methyl difluoroacetate (3.0 eq) was added dropwise and the reaction mixture was heated at 40 C overnight. Upon completion of the reaction, the reaction mixture was evaporated to dryness under vacuum and the residue was subjected to FCC (AcOEt) to afford pure conjugate **20** as a pale yellow oil (95%).[3]

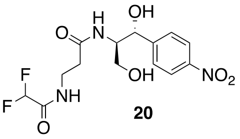

20

Rf (EtOAc, Merck): 0.1, **1H-NMR** (600 MHz, DMSO-d_6) δ 8.59 (s, 1H), 8.16 (d, 2H, *J* = 8.7 Hz), 7.64 (d, 1H, *J* = 9 Hz), 7.55 (d, 2H, *J* = 8.7 Hz), 5.78 (d, 1H, *J* = 4.8 Hz), 4.99 (s, 1H), 4.79 (t, 1H, *J* = 4.8 Hz), 3.97 (d, 1H, *J* = 8.4 Hz), 3.54 – 3.50 (m, 1H), 3.14 (q, 2H, *J* = 6.8 Hz), 2.32 – 2.16 (m, 2H) ; **13C-NMR** (DMSO-d_6,151 MHz) δ 170.3, 162.4, 152.4, 146.9, 127.9, 123.0, 108.9, 69.9, 60.7, 56.1, 36.3, 34.8; **ESI-MS** (30eV) m/z: 384.85 $[M+Na]^+$

E. Synthesis of analogue 21

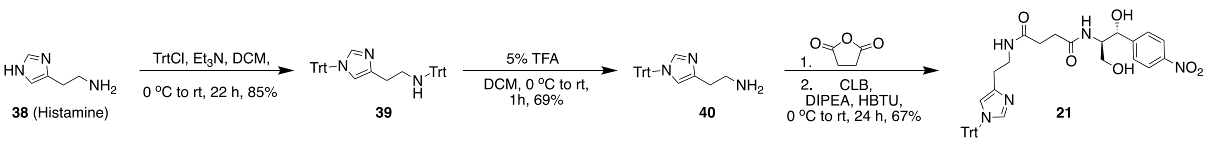

Scheme 5. Synthesis of analogue 21 from commercially available Histamine.

Bis-Trityl-protection of Histamine

To an ice-cold solution of the commercially available histamine (1.0 eq) in DCM (0.4 M), were added sequentially and dropwise Et_3N (5.0 eq) and a solution of TrtCl (2.5 eq) in DCM (1.0 M). Then, the reaction mixture was allowed to warm up at room temperature. After overnight stirring (monitored by TLC), it was diluted with DCM, washed with 5% aqueous citric acid, H_2O and brine, dried over Na_2SO_4, filtered and concentrated under reduced pressure. The residue was subjected to FCC (PhMe/AcOEt 9:1) to afford upon solvent removal **39** as a yellow solid (85%).

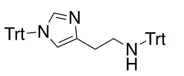

39

R_f (PhMe/EtOAc 9:1, Merck): 0.2; mp 200-203°C; ^{1}**H-NMR** ($CDCl_3$, 600 MHz) *δ* 7.39 – 7.11 (m, 30H,), 6.51 (s, 1H), 2.74 (t, 2H, *J* = 6.3 Hz), 2.40 (t, 2H, *J* = 6.3 Hz), 2.36 (s, 1H) 2.02 (s, 1H); ^{13}**C-NMR** ($CDCl_3$,151 MHz) *δ* 146.2, 142.5, 138.4, 129.7, 129.0, 128.7, 128.2, 128.0, 127.9, 127.7, 126.1, 125.3, 118.5, 75.1, 70.8, 42.8, 42.3, 29.3; **ESI-MS** (30eV) m/z: 618.46 $[M+Na]^+$

Selective Trt-deprotection of the primary amine of compound 39

To a solution of 1**5** (1.0 eq) in DCM (0.34 M), a solution of 5% TFA (2.0 eq) in DCM was added dropwise at room temperature. After 1 h the reaction mixture was diluted with DCM and washed with 5% aqueous $NaHCO_3$, H_2O and brine. After being dried over Na_2SO_4, the organic extract was filtered and evaporated to dryness under vacuum. The residue was subjected to FCC (DCM/MeOH/NH_3 95:5:0.1) and providing the pure product **40** as a pale yellow oil (69%).

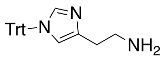

40

R_f (DCM/MeOH/NH_3 95:5:0.1, Merck): 0.4; ^{1}**H-NMR** ($CDCl_3$, 600 MHz) *δ* 7.35 – 7.27 (m, 15 H), 6.59 (s, 1H), 3.04 (t, 2H, *J* = 6.4 Hz), 2.71 (t, 2H, *J* = 6.4 Hz), 1.26 (s, 1H); ^{13}**C-NMR** ($CDCl_3$,151 MHz) *δ* 142.4, 138.6, 129.7, 128.0, 127.9, 127.9, 127.2, 118.5, 75.2, 41.6, 30.4

Synthesis of N_{im}-Trt-Histamine-succinyl-CLB (21)

To an ice-cold solution of **40** (1.0 eq) in DMF (0.4 M), succinic anhydride (1.2 eq), and dropwise DIPEA (1.2 eq) were added. The reaction mixture was stirred at room temperature overnight and it was monitored by TLC. Then, the mixture was cooled at 0° C and followed addition of CLB (1.1 eq), HBTU (1.1 eq) and DIPEA (1.2 eq) dropwise. After overnight stirring at room temperature, it was evaporated to dryness under vacuum and diluted with DCM. The organic phase was washed with 5% aqueous $NaHCO_3$, H_2O and brine, dried over Na_2SO_4, filtered and evaporated to dryness under vacuum. The residue was subjected to FCC (DCM/MeOH 9:1) and gave the pure product **21** as a pale yellow oil (67%).

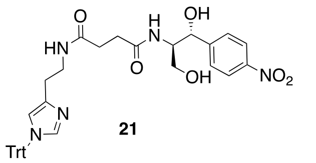

21

R_f (DCM/MeOH 9:1, Merck): 0.1; ^{1}**H-NMR** ($CDCl_3$, 600 MHz) *δ* 8.14 (d, 2H, *J* = 8.5 Hz), 7.94 (s, 1H), 7.55 (d, 2H, *J* = 8.5 Hz), 7.39 – 7.28 (m, 9H), 7.10 – 7.02 (m, 6H), 6.75 (s, 1H), 5.13 (s, 1H), 4.03 (s, 1H), 3.85 (d, 1H, *J* = 11 Hz), 3.73 (d, 1H, *J* = 10.6 Hz), 3.65 (d, 1H, *J* = 10.2 Hz), 3.52 – 3.47 (m, 2H), 2.86 (s, 2H), 2.17 (s, 1H), 2.08 (s, 1H), 2.04 (s, 1H).; ^{13}**C-NMR** ($CDCl_3$,151 MHz) *δ* 147.2, 129.6, 128.7, 127.9, 123.4, 99.1, 81.9, 72.9, 29.7; **ESI-MS** (30eV) m/z: 670.29 $[M+Na]^+$

F. Synthesis of Reduced-urocanic acid-CLB analogue 22

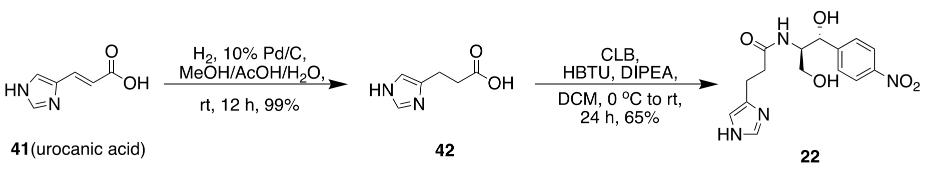

Scheme 6. Synthesis of analogue 22 from commercially available urocanic acid.

Catalytic hydrogenation of urocanic acid

A solution of the commercially available urocanic acid (1.0 eq) in MeOH/AcOH/H_2O 1/0.025/0.0125 was subjected to hydrogenolysis over 10% Pd/C (0.172 eq) at ambient temperature and pressure for 12h. Thus, the reaction mixture was filtered through Celite and the filter cake was washed several times with methanol. After removal of the solvent under reduced pressure, **42** was obtained and used in the next step without any further purification. **ESI-MS** (30eV) m/z: 141.82 $[M+H]^+$

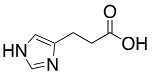

42

Synthesis of analogue 22

To an ice-cold solution of the acid **42** (1.0 eq) in DCM (0.8 M), HBTU (1.2 eq) CLB (1.2 eq), and dropwise DIPEA (1.2 eq) were added. The reaction mixture was allowed to gradually warm up to room temperature. After overnight stirring at room temperature, it was diluted with DCM, washed with 5% aqueous citric acid, H_2O and brine. The organic phase was dried over Na_2SO_4, filtered and evaporated to dryness under vacuum. The residue was subjected to FCC (PhMe/EtOAc 1:9) to give the pure product 22 as a colourless oil (60%).[1,2]

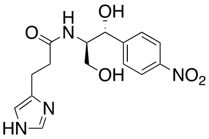

22

R_f (PhMe/EtOAc 1:9, Merck): 0.1; ^{1}H-NMR ($CDCl_3$, 600 MHz) δ 8.22 (d, 2H, J = 8.7 Hz), 7.95 (d, 1H, J = 8.4 Hz), 7.69 (d, 2H, J = 8.6 Hz), 7.41 – 7.38 (m, 1H), 7.34 (m, 1H,), 5.85 (d, 1H, J = 7.7 Hz), 5.23 (d, 1H, J = 8.5 Hz), 4.57 – 4.54 (m, 2H), 4.46 (dd, 2H, J = 3.9 & 11.5 Hz), 3.96 (dd, 1H, J = 3 & 11.5 Hz), 3.41 (s, 1H), 3.21 (s, 1H), 2.17 (s, 2H); ^{13}C-NMR ($CDCl_3$,151 MHz) δ 157.6, 148.4, 143.2, 143.0, 128.7, 128.6, 127.3, 125.0, 123.8, 120.3, 108.6, 90.7, 61.6, 55.3, 36.2, 29.7, 22.7, 14.1; ESI-MS (30eV) m/z: 357.5 $[M+Na]^+$

References

1. Giannopoulou PC, Missiri DA, Kournoutou GG, et al. New Chloramphenicol Derivatives from the Viewpoint of Anticancer and Antimicrobial Activity. *Antibiot (Basel, Switzerland)*. 2019; 8(1):9. doi:10.3390/antibiotics8010009

2. Tereshchenkov AG, Dobosz-Bartoszek M, Osterman IA, et al. Binding and Action of Amino Acid Analogs of Chloramphenicol upon the Bacterial Ribosome. *J Mol Biol*. **2018**; 430(6):842-852. doi:https://doi.org/10.1016/j.jmb.2018.01.016

3. Seiple IB, Mercer JAM, Sussman RJ, Zhang Z, Myers AG. Stereocontrolled synthesis of syn-β-Hydroxy-α-amino acids by direct aldolization of pseudoephenamine glycinamide. *Angew Chem Int Ed Engl*. **2014**; 53(18):4642-4647. doi:10.1002/anie.201400928

4. Papaioannou D, Athanassopoulos C, Magafa V, et al. Facile Preparation of the 1-Hydroxybenzotriazolyl Ester of N-Tritylpyroglutamic Acid and its Application to the Synthesis of TRH, [D-His2]TRH and Analogues Incorporating cis- and trans-4-Hydroxy-L-proline. *Acta Chem Scand - ACTA CHEM SCAND*. **1995**; 49:103-114. doi:10.3891/acta.chem.scand.49-0103
